# The Role of Autophagy in Long-term Effects of Spinal Cord Injury: Does Exosome Change the Autophagy Process and Vice Versa?

**DOI:** 10.34172/apb.025.43284

**Published:** 2025-07-08

**Authors:** Naeimeh Akbari-Gharalari, Abbas Ebrahimi-Kalan, Zeinab Aliyari-Serej, Hamid Soltani Zangbar, Maryam Ghahremani-Nasab, Yahya Yahyavi, Ayyub Ebrahimi, Negar Aghaei, Farshad Nezhadshahmohammad

**Affiliations:** ^1^Neurophysiology Research Center, Cellular and Molecular Medicine Research Institute, Urmia University of Medical Sciences, Urmia, Iran; ^2^Department of Neuroscience and Cognition, Faculty of Advanced Medical Sciences, Tabriz University of Medical Sciences, Tabriz, Iran; ^3^Neurosciences Research Centre (NSRC), Tabriz University of Medical Sciences, Tabriz, Iran; ^4^Department of Applied Cell Sciences, Faculty of Advanced Medical Sciences, Tabriz University of Medical Sciences, Tabriz, Iran; ^5^Stem Cells and Regenerative Medicine Institute, Tabriz University of Medical Sciences, Tabriz, Iran; ^6^Department of Molecular Medicine, Faculty of Advanced Medical Sciences, Tabriz University of Medical Sciences, Tabriz, Iran; ^7^Department of Molecular Biology and Genetic, Faculty of Arts and Sciences, Halic University, Istanbul, Turkey; ^8^Faculty of Medicine, Tabriz Medical Sciences, Islamic Azad University, Tabriz, Iran; ^9^Department of Mining Engineering, Faculty of Engineering, Urmia University, Urmia, Iran

**Keywords:** Autophagy, Exosome, Signaling pathways, Spinal cord injury

## Abstract

This review explores the synergistic effects of exosomes and autophagy in mitigating spinal cord injury (SCI) while elucidating the underlying molecular mechanisms. We evaluate current literature on the roles of exosomes and autophagy in SCI, highlighting key findings from both in vitro and in vivo studies. Previous research has demonstrated the contributions of these processes to cellular responses under SCI-related conditions. Additionally, animal models have provided insights into the therapeutic potential of modulating exosome and autophagy pathways. The overview discusses the activation of mTOR signaling and autophagy-related proteins, emphasizing their impacts on inflammation and axonal integrity. We identify a synergistic mechanism in which exosome-mediated cargo delivery and autophagy modulation work together to mitigate the effects of SCI. The regulation of the mTOR pathway and autophagy-related proteins is crucial for reducing inflammation and preserving axonal integrity. These findings underscore the therapeutic potential of targeting exosomes and autophagy for SCI treatment. The collaborative actions of these cellular processes present promising therapeutic avenues for SCI and possibly other neurological disorders. This review underscores the necessity for further studies to unravel the molecular intricacies and to translate these findings into clinical applications for SCI patients.

## Introduction

 A disruption to the spinal cord resulting from traumatic injury can lead to temporary or permanent disruptions in the cord’s normal sensory, motor, and autonomic function, resulting in severe neurological deficits and physical disabilities. Such a condition is known as spinal cord injury (SCI).^1^ The incidence rate of SCI varies across different countries and regions, ranging from 8 to 906 cases per million individuals, with the highest prevalence observed in males under the age of 30.^2^ SCI has the potential to significantly impact an individual’s quality of life by disrupting normal bodily functions, including bowel, bladder and sexual functions, respiration, and hormone release. Consequently, the effects of SCI can alter an individual’s lifestyle.^3^ Following spinal cord damage, the regrowth of axons in the spinal cord is impeded by various factors, including inhibitory elements present in glial scars, myelin, and a deficiency of neurotrophic and nerve growth factors. This impediment results in the inability of neurons to regrow spinal cord axons.^4^ Various therapeutic modalities are available for the treatment of SCI, including pharmaceutical interventions, surgical procedures, and rehabilitation programs. These interventions are integral components of the conventional SCI management plan, aimed at preventing further loss of function and promoting functional recovery.^5^ Apart from the conventional treatment modalities, researchers have made significant strides in investigating the potential therapeutic applications of extracellular vesicles, particularly exosomes.^6^ These vesicles have been implicated in the pathogenesis of several diseases and are critical for mediating intercellular communication.^7^ Multiple techniques have been suggested to identify the interactions between exosome-receiving cells, including attachment or merging with the recipient cell membrane, transcytotic uptake by the receiving cells, and cellular connections facilitated by receptor-ligand associations.^8^ The transfer of various molecules, including noncoding RNA, coding RNA, DNA, signal transduction molecules, and proteins, facilitates communication between cells. This process supports the transportation of membrane receptors, which can trigger target cells, and proteins, which can induce epigenetic changes in recipient cells. These mechanisms play critical roles in the intercellular communication of the central nervous system (CNS), enabling communication between neurons and glia, promoting neuronal regeneration and proliferation, and regulating immune responses.^9^ The maintenance of intracellular homeostasis is thought to rely on the interplay between exosomes and autophagy.^10^ Autophagy is a widespread metabolic process that supports cellular survival in most eukaryotic cells. When confronted with various stressors such as hypoxia, starvation, and endoplasmic reticulum stress, autophagy can break down protein complexes and other constituents into organic molecules within the cytoplasm as a self-preservative mechanism.^11^ Autophagy also aids in preserving intracellular equilibrium by removing denatured proteins and damaged organelles. Inadequate or inappropriate autophagy under chronic or acute stress has been strongly linked to the development of several diseases, including malignancies, neurological disorders, fatty acid metabolism disorders, and immunological pathologies. This can result in the accumulation of toxic substances and ultimately lead to cell death.^12^ Autophagy is increasingly recognized as a two-edged sword in the context of SCI: excessive or dysregulated autophagy leads to tissue damage and cell death, while moderate activation may provide neuroprotective effects by removing cytotoxic material and enhancing cell survival. Recent studies have demonstrated the molecular and clinical significance of autophagy pathway modulation in neurotrauma research by suggesting that it may have therapeutic potential for reducing neuronal damage and fostering functional recovery following SCI.^13,14^ This review aims to identify the collaborative relationship between exosomes and autophagy in alleviating pathological conditions associated with SCI, as well as to elucidate the changes in autophagy, the effects of exosomes, and the signaling pathways involved. By acquiring a deeper understanding of the interplay between exosomes and autophagy, it is expected that novel therapeutic strategies for SCI patients can be developed.

## Mechanism and signaling pathways of autophagy

 The process of lysosomal degradation of cellular components within the cell is known as “autophagy” and is considered a stress response. Within the autophagic system, all durable proteins and organelles are recycled. Autophagy encompasses various types, such as macro-, micro-, and chaperone-mediated autophagy (CMA).^15^ Macro-autophagy is the primary type of autophagy associated with multiple physiological functions, including intracellular quality control, development, and cell death. It involves the formation of vesicles that can occupy a significant portion of the cytoplasm. In contrast, micro-autophagy and CMA are not associated with major morphological changes in vesicular compartments.^16^ The initial stage of the autophagic process involves the formation of autophagosomes, which subsequently degrade cargo with the aid of hydrolytic enzymes. These autophagosomes are double-membrane vesicles that transport cargo to lysosomes. Subsequent to the formation of autophagosomes, the outer membrane of the autophagosome fuses with the lysosomal membrane, leading to the degradation of the contents of the vesicle. The biomolecules resulting from this degradation undergo recycling and are returned to the cytoplasm for reuse.^17^ Autophagy processes such as pexophagy, mitophagy, reticulophagy, and ribophagy are defined based on the type of cargo being degraded.^18^ The regulation of autophagy involves several signaling pathways. For example, phosphoinositide 3-kinase (PI3K) and enzymes responsible for generating Phosphatidylinositol 3-phosphate (PtdIns-3P) play a crucial role in autophagic pathways. While Class III PI3K is essential for initiating autophagosome formation, Class I PI3K has an inhibitory effect on the process.^19^ The expression of Beclin I is also associated with the formation of autophagosomes. Studies have demonstrated that Beclin I and Class III PI3K work in conjunction to interface with constituents of the B-cell lymphoma 2 (Bcl-2) complex.^20^ MTOR is a serine/threonine protein kinase that serves as a significant regulator of autophagy. It is a downstream kinase of PI3K that acts to inhibit apoptotic signals. A comprehensive understanding of these pathways is necessary to comprehend pathological conditions. Autophagy is considered a double-edged sword,^21^ as it is unclear whether inhibition or induction is more efficacious, and therefore warrants further discussion.

## Autophagy and neurodegenerative disease

 Neurons within the nervous system transfer organelles and proteins via intercellular communication through axons. Synapses with a high density of mitochondria and ribosomes are particularly vulnerable to impairment of the autophagy process. As neurons are incapable of replication, they are susceptible to the accumulation of damaged organelles and proteins.^22^ The function of autophagy as a degradation system for removing aggregated and ubiquitinated proteins, as well as the dysregulation of the autophagic pathway in neurodegenerative disorders, are both subjects of investigation.^23^ Autophagy is activated in response to pathological conditions in the CNS, including stroke, SCI, infection, and traumatic brain injury (TBI).^24^ The autophagic pathway mitigates disease progression and supports the neuroprotective role of autophagy by removing protein aggregates.^25^ As such, comprehending the importance of autophagy pathways in neurodegenerative conditions and the effects of drugs (listed in Table 1) could prove beneficial to future investigations on the treatment of SCI.

**Table 1:** Autophagy signaling pathways and medications that reduce aggregated molecules in neurodegenerative diseases.

**Disorder**	**Aggregated molecules**	**Drug**	**Signaling pathway**	**Effect on autophagy**	**Reference**
MS	Fibronectin	Haloperidol/ Clozapine	AMPK/mTOR/ ULK1	Inhibition	^26,27^
AD	β-amyloid plaques/Tau	Carbamazepine	(mTOR)-independent	Induction	^28^
HD	Huntingtin protein with abnormal glutamine repeat	Lithium	(mTOR)-independent	Induction	^29,30^
PD	α-Synuclein/Lewy body	Rapamycin	mTOR	Induction	^31^
ALS	TDP-43/FUS	Lithium/ Valproate	(mTOR)-independent	Induction	^32,33^
Stroke	TDP43/hnRNPA/PSF/SFPQ/p54/ NONO/…	Minocycline/ Melatonin	PI3K/AKT/mTOR	Inhibition	^34-36^
FTD	TDP-43/FUS	Rapamycin	mTOR	Induction	^37-39^
SCI	SQSTM1	Simvastatin	mTOR	Induction	^40,41^

## Pathophysiology of SCI

**Figure 1:**
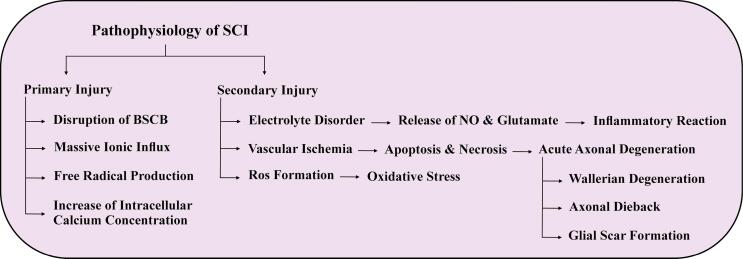
Pathophysiology of SCI

 Spinal cord injury, a debilitating neurological disorder, is associated with multiple consequences, such as physical dependence, morbidity, psychological distress, and financial burden.^42^ However, existing treatment methods offer only temporary relief, and thus, complete recovery is not anticipated. The term “primary injury” denotes the initial phase of damage, which encompasses bone fragments, torn spinal ligaments, bleeding, rupture of glial membranes, loss of neuronal parenchyma, and damage to the axonal network.^43^ The secondary injury, which is triggered by the primary injury, results in increased reactive oxygen and glutamate levels, exacerbates chemical and mechanical damage to the spinal tissues and induces neuronal excitotoxicity due to the accumulation of intracellular calcium.^1^ Furthermore, autophagy serves as a recycling mechanism that eliminates unnecessary proteins and organelles by enhancing autophagosomes and lysosomal pathways and is also a critical element in regulating cell death.^44^ However, if autophagosomes and lysosomes are inappropriately activated during SCI, it can lead to rapid cell death. Researchers have investigated the series of events that take place in the pathophysiology of SCI (as shown in Figure 1), encompassing various multicellular and multimolecular interactions and potential therapeutic interventions. Recent studies have focused on the role of exosomes and their effects on autophagy.

## Autophagy in SCI

 The succession of autophagy-related events following the primary physical trauma to the spinal cord in instances of SCI carries substantial significance. The level of autophagy flux, which is contingent on the type and placement of the SCI lesion, may either increase or decrease. Notably, in cases of mild injury, there may be a defensive effect due to elevated levels of autophagy flux.^45^ However, the secondary injury that gradually develops following SCI hinders the autophagy-lysosome pathway. Under stressful conditions, autophagy is usually maintained at low levels but is activated to promote cell survival through the unregulated breakdown of misfolded proteins.^46^ Although autophagy is typically a survival mechanism, excessive autophagic activity can lead to cell death, which is distinct from apoptosis.^47^ Following SCI, autophagy is upregulated in both neurons and glial cells, indicating its crucial role in mitigating injury-induced damage.

###  Autophagy in neural cells after SCI

 Autophagy, the process of cellular self-digestion, is commonly observed in neurons after SCI and typically increases within 24 hours of the injury. However, previous studies on autophagy in SCI have largely either not identified the type or location of neurons being investigated, or have focused solely on motor neurons in the ventral horn.^48^ Recent research has demonstrated that there is a greater accumulation of Light Chain 3 (LC3) and Sequestosome1 (SQSTM1) proteins in motor neurons located in the ventral horn compared to sensory neurons in the dorsal horn, despite the latter being closer to the site of injury.^45,49^ This suggests that motor neurons may be more vulnerable to disruptions in autophagy flux, although dorsal sensory neurons can also be affected, as seen in neuropathic pain models involving spinal nerve ligation. Furthermore, autophagosome accumulation has been observed in both neuronal cell bodies and axons following SCI.^13^ Elevated autophagy flux may aid in the removal of dysfunctional organelles, thereby safeguarding cells from additional damage. Indications supporting this proposition involve the detection of autophagosome buildup, including damaged mitochondria and heightened Bcl2 Interacting Protein 3 (BNIP3) levels, both of which suggest the induction of mitophagy, after SCI. Autophagy could also contribute to the elimination of harmful ubiquitin-positive protein aggregates formed following injury and furnish essential building blocks and energy for the commencement of reparative mechanisms subsequent to trauma.^50^

 Inhibition of autophagy flux due to SCI is likely to contribute to neuronal cell death. Markers of both apoptotic and non-apoptotic cell death mechanisms indicate that inhibition of autophagy flux following SCI may initiate and/or amplify multiple cell death pathways. However, a definite causal relationship between autophagy flux inhibition and neuronal cell death in these models has yet to be established. In SCI, a correlation has been observed between the inhibition of autophagy flux and endoplasmic reticulum (ER) stress in neurons. Although the mechanisms underlying ER stress as a component of secondary injury after trauma are not fully understood, autophagy is commonly increased and can function as a protective mechanism in response to ER stress. Therefore, inhibiting autophagy flux after injury could worsen ER stress, leading to the induction of apoptosis.^11,51^ It should be highlighted that inhibiting autophagy flux following trauma may only have a transient impact. Therefore, in the early phases after SCI, the accumulation of autophagosomes may contribute to neuronal cell death. However, at later time points when autophagic flux is reinstated, autophagy may exert a protective effect. It remains unclear how autophagy flux is restored after SCI, but the timing corresponds to a rise in lysosomal production. This may compensate for the initial harm inflicted on lysosomal organelles. Alternatively, the heightened flux could be due to the demise of affected cells.^13^

 Preventing the flow of autophagy may lead to neuronal cell death through non-apoptotic means following injury. Necroptosis, a form of regulated necrosis that relies on Receptor Interacting Serine/Threonine Kinase (RIPK1/RIPK3), is another mode of cell death that may be influenced by autophagy. Inhibiting necroptosis has been shown to improve functional outcomes after SCI, suggesting this pathway’s involvement in secondary injury. Recent findings suggest that autophagosomes may act as platforms for the assembly of necrosomes, which promote necroptosis and contain RIPK1, RIPK3, and Autophagy-related 5 (ATG5). As a result, autophagosome accumulation following SCI may also contribute to necroptosis.^52^

###  Autophagy in non-neural cells after SCI

 Autophagy is elevated in multiple non-neural cell types within the spinal cord following SCI. Astrocytes expressing glial fibrillary acidic protein accumulate autophagosomes, and increased autophagy has also been reported in oligodendrocytes after injury.^53^ Similarly, elevated LC3 levels have been observed in microglia, with the extent of autophagosome accumulation in all glial cells generally greater near the injury site.^48^ Notably, autophagosome buildup in glial cells occurs later than in neurons, typically starting around day 3 post-injury, suggesting differential sensitivities or distinct autophagic mechanisms between these cell types.^54^ Oligodendrocyte loss is a common and critical aspect of secondary injury after SCI, and as in neurons, autophagy flux—whether initiated or obstructed—may influence their survival or death.^55^ Supporting this, a rat model of myelin mutation demonstrated that autophagy promotes oligodendrocyte precursor survival and myelin formation, indicating a potentially similar function after SCI.^56^ Moreover, autophagy is implicated in regulating inflammatory responses via the nuclear factor kappa B (NFκB) pathway; the interaction between SQSTM1 and tumor necrosis factor receptor associated factor 6 (TRAF6) can activate NFκB, suggesting autophagy might modulate inflammation in microglia and infiltrating macrophages post-injury.^57,58^ However, further studies are required to clarify how autophagy impacts glial survival and function after SCI.^59^ Experimental models reveal that autophagic responses in non-neural cells are both cell-type specific and temporally distinct. Around 7 days post-injury, astrocytes and oligodendrocytes show marked autophagosome accumulation; reactive astrocytes exhibit increased autophagy initiation, whereas oligodendrocytes accumulate autophagic structures primarily due to impaired autophagic flux.^60^ In myelinating glia, autophagy plays essential roles beyond basic turnover, contributing to myelin formation through membrane turnover and cytoplasm removal, and facilitating remyelination after SCI. It also supports tissue remodeling and phagocytosis, processes crucial for homeostasis and repair in the injured spinal cord.^61^ Recent research further emphasizes the importance of autophagy in microglia. In a rat T10 contusion model, treatment with Fisetin, a natural anti-inflammatory compound, enhanced autophagic activity in pro-inflammatory microglia via the AMPK–mTOR pathway. This activation reduced pro-inflammatory markers (CD68, iNOS) and cytokines (IL-6, TNF-α), attenuating neuroinflammation and promoting neurological recovery. Inhibition of autophagy partially reversed these benefits, underscoring microglial autophagy as a key modulator of secondary injury and a potential therapeutic target.^62^

###  Autophagy signaling pathways after SCI

 Following SCI, autophagy can be initiated by various factors such as the accumulation of impaired organelles and proteins, and the release of inflammatory cytokines and chemokines. The regulation of autophagy in response to these factors is complex and involves numerous signaling pathways. The expression of the LC3 protein, which is widely used as a marker of autophagy, varies depending on several factors, including cellular location, cell type, and the duration after SCI. In neurons, LC3 expression displays a slight elevation two hours after injury, which gradually declines one day later. However, comparable increases in LC3 expression are evident three days after injury, particularly in astrocytes, indicating that autophagy reaches its maximum level seven days after injury. Furthermore, the ratio of LC3-II to LC3-I in the spinal cord considerably increases on day 3, peaks on day 7 after SCI, and then rapidly declines over the subsequent 21 days. These findings suggest that autophagy is a dynamic process that is regulated in a time-dependent manner after SCI.^11,48,63^

 The production of autophagosomes is reliant on Beclin-1, a component of the class III PI3K complex.^19^ Beclin-1 is normally suppressed by Bcl-2, but during periods of stress, Beclin-1 is liberated from Bcl-2, leading to the activation of vacuolar protein sorting 34 (Vps34).^13^ Active Vps34 generates autophagosomes through the production of Phosphatidylinositol 3-phosphate (PtdIns3P). The Beclin-1/Bcl-2-interacting protein is thought to be a stimulator of autophagy, and Jun N-terminal kinase (JNK) activation triggers the phosphorylation of Bcl-2 and the liberation of Beclin-1.^64^ Additionally, autophagy can regulate apoptosis by modulating Bcl-2 and reducing Bcl-2-associated X (Bax). Beclin-1 expression has been identified in both neurons and glia.^65^ Beclin-1 expression reaches its maximum level 24 hours after injury, according to a study by Zhang Q. et al., while Bcl-2 levels were significantly diminished at the same time point.^66^ These findings suggest that Beclin-1 and Bcl-2 may play a role in both autophagy and apoptosis following SCI. JNK1 interacts with Beclin-1 to phosphorylate Bcl-2, which inhibits autophagy, while the PI3K/Vps34 complex, with Beclin-1 as a subunit, is essential for autophagosome formation. Overexpression of nitric oxide (NO) and reactive oxygen species (ROS) can impede the JNK1/Bcl-2/Beclin-1 pathway that is crucial for autophagy regulation, while ROS can also activate the autophagy-related gene 4 (ATG4), which promptly initiates autophagy.^67^ MTOR is an upstream mediator that regulates JNK, and only mTORC1 plays a direct role in autophagy regulation.^68^ AMP-activated kinase (AMPK), phosphatase and tensin homolog (PTEN), and tuberous sclerosis complex1/2 (TSC1/2) are considered positive regulators, while Akt is an inhibitor of autophagy. AMPK is the primary cellular energy sensor that is activated by an increase in AMP levels, which is necessary for autophagy initiation. When energy levels drop, AMPK limits mTOR activity by permitting access to the TSC1/2 system, a negative regulator of mTORC1.^69^ AMPK can also initiate autophagy by directly activating Unc-51-like kinase1 (ULK1), which triggers autophagy. The ATG1/ULK1 complex is believed to initiate the autophagic cascade according to most studies.^13^ Activation of AMPK can also elevate JNK1 when Beclin-1 and Bcl-2 dissociate to form the Vps34 complex and induce autophagy.^16^

 Cells generate heat shock proteins (HSPs) in stress response, which can be identified by their molecular weight. HSPs are involved in CMA, which facilitates the entry of cytosolic proteins into lysosomes. Following SCI, ependymal, endothelial, and microglial cells frequently produce HSPs to protect motor neurons from further damage.^70^ Various subtypes of HSPs are utilized in neuroprotection strategies. For example, to prevent neuronal demise, a complex is formed between Akt and HSP25.^71^ Unlike HSP27, which safeguards neurons by operating upstream of caspase-3 and downstream of cytochrome C released from mitochondria, HSP90 affects the P2X7 receptor and stimulates the death-inducing Fas cascade.^72^ HSP70 acts as a chaperone and prevents protein denaturation and harmful aggregation cascades through various folding and holding mechanisms, thereby preventing cell death and protecting neuronal cells from injury. Although HSP70 is rarely found in healthy individuals, it is predominantly present in SCI, and its expression increases following injury. The function of HSP70 is influenced by the level of HSP expression and the intensity of stimulation.^73^ These findings suggest that HSPs play a crucial role in neuroprotection following SCI and underscore the importance of further research to develop targeted therapies that can leverage the protective mechanisms of HSPs.

 Fascinating research explores the possibility of transforming damaged spinal cord cells into induced pluripotent stem cells (iPSCs) to facilitate neural regeneration by manipulating autophagy. In a recent study, researchers introduced a novel method using specific reprogramming factors (Sox2, Oct4, Klf4, and c-Myc, collectively referred to as 4F) to induce autophagy while inhibiting the mTORC1 pathway during the conversion of fibroblast cells into iPSCs. The study revealed that the 4F factors work together to suppress mTORC1 and remodel cells, but they have varying effects on autophagy-related genes: Klf4 and c-Myc increase their expression, while Sox2 and Oct4 reduce it. This balance between mTORC1 suppression and autophagy induction shows promise for treating spinal cord injuries and potentially regenerating the spinal cord. However, further research is needed to confirm and fully understand these findings before clinical application.^74,75^

 The potential of autophagy to exhibit both protective and pathogenic effects is evident, and the precise impact of autophagy on the different stages of SCI remains unclear. Therefore, future investigations should aim to comprehensively elucidate the mechanisms involved in autophagy during SCI, to develop a versatile therapy that can target various cell types and pathways throughout the degenerative phase of SCI, based on the significance of autophagy in this condition.

## Exosomes features

 The studies conducted by Harding et al. led to the discovery of exosomes, which are extracellular vesicles that play a role in various physiological and pathological processes and facilitate intercellular communication.^76^ Exosomes have a diameter of 30–150 nm, as shown by electron microscopy.^72^ The lipid content of exosomes mainly includes sphingolipids, cholesterol, and glycerophospholipids, along with signaling proteins such as Catenin, Delta-like 4, IL-1, and TNF-α.^77^ In addition to lipids and proteins, exosomes contain mRNA, miRNA, and mtDNA, which can be utilized for therapeutic purposes in transferring data.^7^ Exosomes are capable of performing a variety of functions, depending on their source cells, which can include macrophages, rhabdomyosarcoma, osteoclasts, mesenchymal stem cells, and exosomes derived from platelet-rich plasma (PRP).^78^ To evaluate the properties of exosomes such as size, surface charge, shape, and density for biological purposes and cargo delivery, various techniques have been employed, including dynamic light scattering (DLS), nanoparticle tracking analysis (NTA), resistive pulse sensing, electron microscopy, atomic force microscopy (AFM), and flow cytometry.^79^ Due to their small size, exosome isolation is challenging and sample-dependent, using methods like ultrafiltration, ultracentrifugation, chromatography, affinity capture, and polymer-based precipitation.^80^

**Figure 2:**
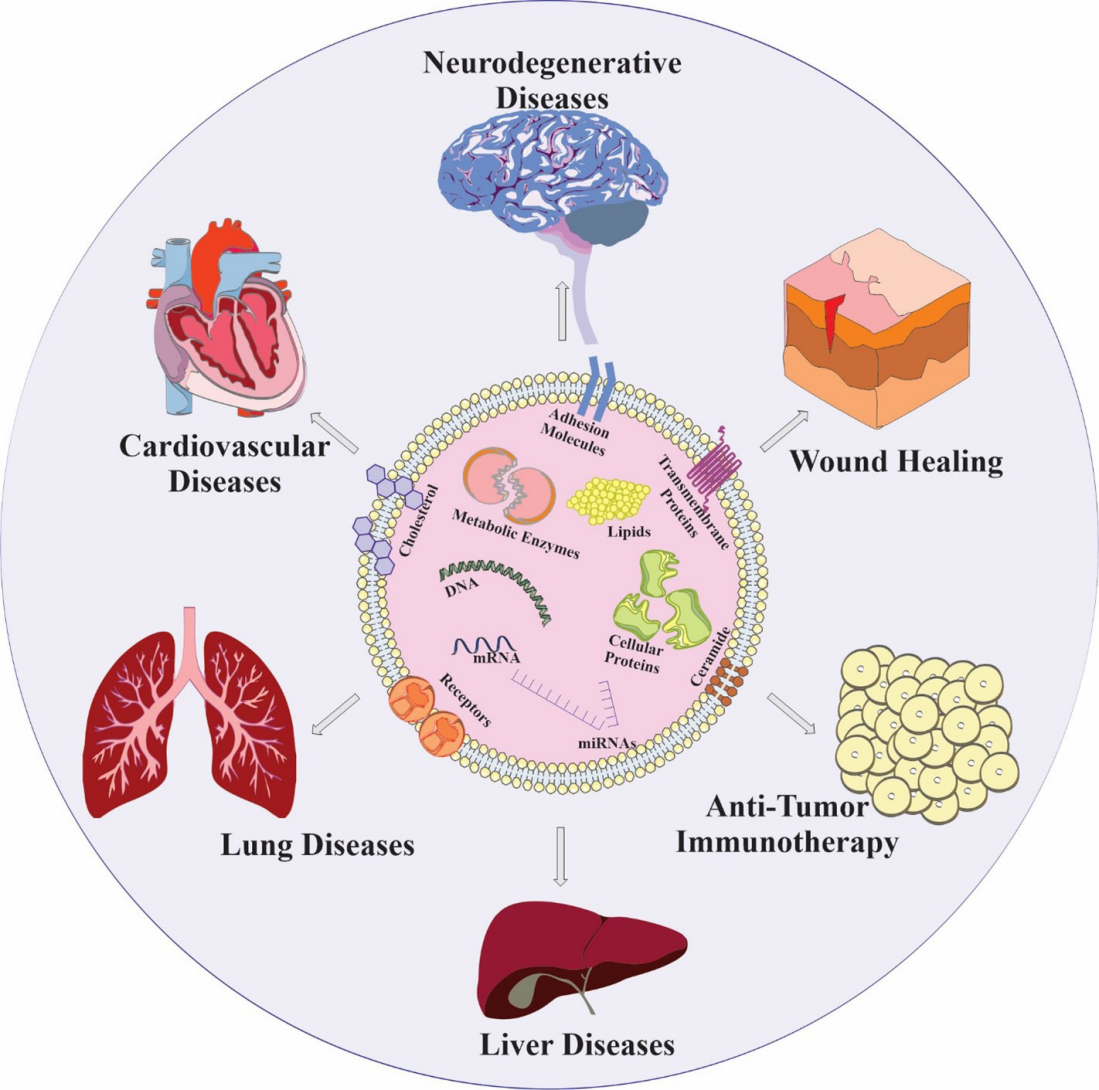
Exosome applications. (AM: Adhesion molecules, TP: Transmembrane proteins, ME: Metabolic enzymes, CP: Cellular proteins)

 Exosomes are a promising cell-free therapy, but their short shelf-life requires preservation methods like freeze-drying, freezing, and spray-drying.^79^ Exosome research faces challenges like inefficient isolation, limited characterization, and lack of specific biomarkers. Distinguishing them from similar structures also complicates analysis. Using proper controls, optimizing conditions, and preventing contamination can improve purity. Their biocompatibility makes them a potential drug delivery tool.^81^ There are several techniques available to load cargo into exosomes, including diffusion across the cell membrane during incubation, endogenous expression for nucleic acids and RNAs, and creation of membrane holes using surfactant, electroporation, and sonication processes. Promoting encapsulation of components into exosomes can also be achieved through extrusion, dialysis, and freeze-thaw during membrane recombination processes.^82^ Modified exosomes have been utilized as therapeutic agents for various conditions, such as bone-related disorders, cancer, skin damage, and neurodegenerative diseases, including those that are difficult to treat due to the blood-brain barrier (BBB). Exosomes have the potential to cross the BBB, making them a promising tool in medicine.^83^ Figure 2 illustrates the various applications of exosomes.

## Exosome application in SCI

 Exosomes have undergone various clinical trials as a treatment option for SCI. However, ongoing preclinical research in this field suggests their potential for further use. Exosomes have demonstrated several capabilities in promoting the restoration of the integrity of the blood-spinal cord barrier (BSCB), angiogenesis, axon regeneration, regulation of inflammatory and immune responses, inhibition of apoptosis, and repair of damaged spinal cord tissue.^84,85^ A major contributor to the deterioration of SCI is the inability of spinal cord microvascular endothelial cells (SCMECs) to adapt. Studies have shown that exosomes derived from neural stem cells can promote the migration, tube formation, and proliferation of SCMECs in mice models. This pro-angiogenic activity was demonstrated to be mediated by exosomal vascular endothelial growth factor (VEGF-A). Additionally, C57 mice models of SCI showed significant improvements in microvascular density, reduction in the extent of the spinal cord cavity, and restoration of motor function.^86^

 Exosomes obtained from bone marrow stromal cells have been found to bind to microglia and inhibit the synthesis and release of complement mRNA and NFκB following SCI.^87^ Also, in vivo studies in mice models have demonstrated that exosomes derived from mesenchymal stem cells obtained from human umbilical cords promote functional recovery following SCI by suppressing inflammatory cytokines, such as TNF-α, macrophage inflammatory protein (MIP-1α), IL-6, and interferon (IFN-γ).^88^ Furthermore, the use of peptide-modified adhesive hydrogel (Exo-pGel) containing exosomes derived from human MSCs has been shown to effectively reduce inflammation and oxidative stress in rat models. As a result, there is significant neuronal regeneration and preservation of urinary tissue.^89^ Injection of MSC-derived exosomes has been found to lead to a significant reduction in the expression of proapoptotic protein BAX and proinflammatory cytokines TNFα and IL-1β, while levels of antiapoptotic protein BCL-2 and anti-inflammatory cytokine IL-10 were increased. Additionally, administration of MSC-derived exosomes resulted in a substantial improvement in angiogenesis in experimental models of SCI in rats.^90^

 Exosomes have been identified as a rich source of VGF, a protein that induces nerve growth. When encapsulated in fibrin gel, exosomes were shown to promote oligodendrogenesis and improve behavioral and electrophysiological performance in a C57 mice model. Upregulation of neuronal markers at the site of injury suggested an increase in neurogenesis.^91^ In a study conducted by Wei Liu and colleagues, exosomes derived from bone mesenchymal stem cells (BMSCs) were found to enhance the proliferation of human umbilical vein endothelial cells while inhibiting the production of nitric oxide by microglia and preventing the activation of A1 neurotoxic reactive astrocytes. The administration of BMSC-exosomes resulted in a reduction in lesion size by almost 60%, a decrease in neuronal apoptosis by roughly 70%, a reduction in glial scar formation by approximately 75%, and an increase in angiogenic tubule formation and average blood vessel density by over 60% in rat models. Moreover, there was an improvement in axonal regeneration by nearly 80%.^92^

 Exosomes were generated by expressing the genes for tumor susceptibility genes 101, CD63, CD81, CD9, and ALG-2-interacting protein X (Alix) (TSG101) in a study investigating their effects on SCI. Exosomes derived from cerebrospinal fluid (CSF) of individuals with SCI were found to enhance neuronal proliferation via the Extracellular signal-regulated kinase (ERK) signaling pathway, while exosomes from normal CSF did not exhibit such effects.^93^ Moreover, the administration of exosomes derived from human pulmonary-MSCs results in the migration and formation of tubes by human umbilical vein endothelial cells (HUVECs). Intrathecal injection of these exosomes into the spinal cord leads to significant enhancements in vessel counts, vessel capacity fractions, and vascular connections in male-mice-SCI models.^94^ Exosome treatment results in a remarkable increase in the population of SOX2 + GFAP + , PAX6 + Nestin + , and SOX1 + KI67 + cells in the spinal cord, leading to the activation of endogenous brain stem/progenitor cells and subsequent proliferation. Sprague-Dawley-SCI rats treated with exosomes also exhibit a notable increase in neurogenesis and a higher percentage of DCX + MAP2 + neurons. Additionally, Exosomes derived from human placental MSCs (hpMSCs) promote the growth of neural stem cells in vitro and elevate the levels of phosphorylated MEK, ERK, and cAMP-response element binding protein (CREB).^95^

 Serum exosomal miR-125b-5p, miR-152-3p, and miR-130a-3p have been identified as distinguishable and detectable diagnostic markers associated with chronic SCI.^96^ Dysregulation of miRNAs has been observed in the spinal cord following SCI, with miR-133b playing a critical role in neurite outgrowth and neuronal differentiation. Infusion of exosomes containing miR-133b improved hindlimb function recovery in rats with SCI, while also reducing lesion size, preserving neuronal cells, and enhancing axon regrowth. The expression of Ras homolog family member A (RHOA), a direct target of miR-133b, was found to be lower in the group treated with miR-133b exosomes. Furthermore, miR-133b exosomes upregulated the expression of signaling pathway proteins such as ERK1/2, Signal Transducer and Activator of Transcription (STAT3), and CREB, which are vital for neuronal survival and axon regeneration.^97^ Also, exosomes derived from MSCs modified with miR-126 have been shown to promote angiogenesis and migration of HUVECs in vitro by inhibiting the expression of sprouty-related EVH1 domain-containing protein 1 (SPRED1) and phosphoinositide-3-kinase regulatory subunit 2 (PIK3R2). Additionally, exosomes generated from MSCs transfected with miR-126 have been demonstrated to reduce apoptosis and enhance functional recovery after SCI in rats.^98^

###  Clinical trials of exosomes in SCI

 Exosomes have been utilized in a total of 311 clinical trials, indicating their potential as a therapeutic tool. However, further research is required to establish their full potential in various clinical settings. In particular, two clinical studies have been conducted on SCI using exosomes. One study, identified as NCT04120272, began recruitment in Seoul, Korea in 2021, to identify biomarkers and risk factors associated with postoperative delirium in elderly patients who have undergone spinal surgery. Meanwhile, a phase I clinical trial has been completed in Rudrapur, Uttarakhand, India, focusing on the intra-discal injection of exosome-enriched PRP for chronic low back pain in individuals aged 18 to 60 years. However, the outcomes of these clinical trials have not yet been reported.

 Exosomes exhibit the unique capacity to target damaged tissue and cross the BSCB due to their nanoscale size. These features make them an attractive option for drug delivery, combining the advantages of both cells and nanotechnology. Nevertheless, certain unknown aspects of their mechanism of action and safety profile must be elucidated before exosomes can be utilized in clinical trials for SCI.

## Exosome and autophagy signaling interactions in SCI

 Autophagy is known to collaborate with different endomembrane systems, signaling pathways, and biomolecule hydrolysis to regulate endocytosis and exocytosis. Recent studies have established that EVs, particularly exosomes derived from the endosomal system, can synergistically work alongside the autophagy process to maintain cellular homeostasis.^99^ The smallest type of EVs, exosomes, originate from the cytoplasmic multivesicular body (MVB), a subtype of the late endosome. These vesicles, released by most cells, carry bioactive substances such as proteins, RNAs, and even DNA strands to facilitate intercellular communication.^100^ Exosomes are also believed to aid in the elimination, breakdown, and recycling of biomolecules, providing support for the notion that autophagy and exosome mechanisms function together to promote cell survival. Indeed, exosomes and autophagy may cooperate in the removal of cellular waste by carefully encapsulating cellular components, merging autophagosomes with MVBs, and disposing of waste materials externally through exosome excretion. This cooperative interaction contributes to the preservation of cellular equilibrium and assumes a pivotal role in cellular responses to diverse stressors and pathological circumstances.^101^

 Several scientific investigations have demonstrated that Schwann cell-derived exosomes (SCDEs) have the potential to promote autophagy and reduce apoptosis, thereby enhancing axonal preservation and motor function recovery following SCI. Additionally, an increase in SCDEs was observed to reduce the expression of the epidermal growth factor receptor (EGFR), inhibit the Akt/mTOR signaling pathway, and stimulate microtubule acetylation and polymerization, indicating that the EGFR/Akt/mTOR pathway is active.^102^ Blocking the PI3K/AKT/mTOR signaling pathway could potentially boost the activation of microglial autophagy and facilitate the polarization of anti-inflammatory microglia.^103^ Furthermore, exosomes originating from peripheral macrophages might play a significant role in the anti-inflammatory process.^31,104^ Another research study demonstrated that BMSC-Exosomes stimulated the production of autophagosomes, increased the expression of autophagy-related proteins LC3IIB and Beclin-1, and prevented the activation of nucleotide-binding domain, leucine-rich family, pyrin domain-containing-3 (NLRP3) inflammasomes. In addition, BMSC-Exosomes reduced the expression of caspase-3, a protein that promotes apoptosis, while increasing the expression of Bcl-2, a protein that inhibits apoptosis. As a result, BMSC-Exosomes promote autophagy and thus play a role in reducing neuronal cell death.^105^

 The administration of NSC-Exosomes led to a reduction in the expression of the proapoptotic protein BAX, the apoptosis effector cleaved caspase-3, and the proinflammatory cytokines TNF-α, IL-1β, and IL-6. Conversely, the antiapoptotic protein Bcl-2 exhibited an increase. NSC-Exosomes contained a significant concentration of miR-374-5p, which facilitated autophagy flux and prevented apoptosis in damaged neurons. The miR-374-5p molecule specifically targeted SKT-4, a protein that safeguards spinal cord cells through exosomes. The elevation of miR-374-5p levels in neuronal exosomes might contribute to the promotion of autophagy and the enhancement of recovery.^106^ Like the previously mentioned research, exosomes that expressed miR-455-5p were noted to improve autophagy and decrease neuronal cell death, thereby demonstrating neuroprotective effects against spinal cord ischemia-reperfusion injury.^107^

 Additional studies have suggested that exosomes originating from huc-MSCs can relieve inflammatory pain by increasing autophagy and decreasing cell death, which is mediated through the miR-146a-5p/TRAF6 pathway.^108^ Exosomes that overexpressed miR-26a selectively targeted and activated the mTOR pathway to reduce excessive autophagy and promote axonal regeneration, thereby facilitating the healing process of SCI.^109^ After experiencing traumatic injury, the administration of exosomes obtained from MSCs induced increased levels of Olig 2 and HSP70 proteins, as well as autophagy-related proteins, in the damaged spinal cords. This resulted in repair and enhanced function by activating autophagy and elevating the levels of survival-related proteins. Furthermore, it has been observed that exosomes can interact with HSPA8 to regulate autophagy.^110^

 Autophagy and exosomes are closely intertwined in cellular processes. Autophagy exerts a significant influence on exosome functionality by actively participating in the early stages of exosome formation and multivesicular body (MVB) development, which are the precursors of exosomes. Autophagy’s role in selectively removing proteins and organelles has dual implications for exosomes. Firstly, it can enhance exosome biogenesis by directing cargo to the endosomal compartment and regulating the contents of exosomes. Secondly, autophagy impacts the entire lifecycle of exosomes, including their release and uptake, as well as the intricate trafficking and fusion events culminating in exosome secretion from cells.^111^ Additionally, autophagy can modify the surface markers of recipient cells, subsequently influencing their ability to internalize exosomes.^12^ This collaborative interplay between autophagy and exosomes underscores their shared upstream regulatory mechanisms and underscores their intertwined functions in cellular processes.^102,112^

**Figure 3:**
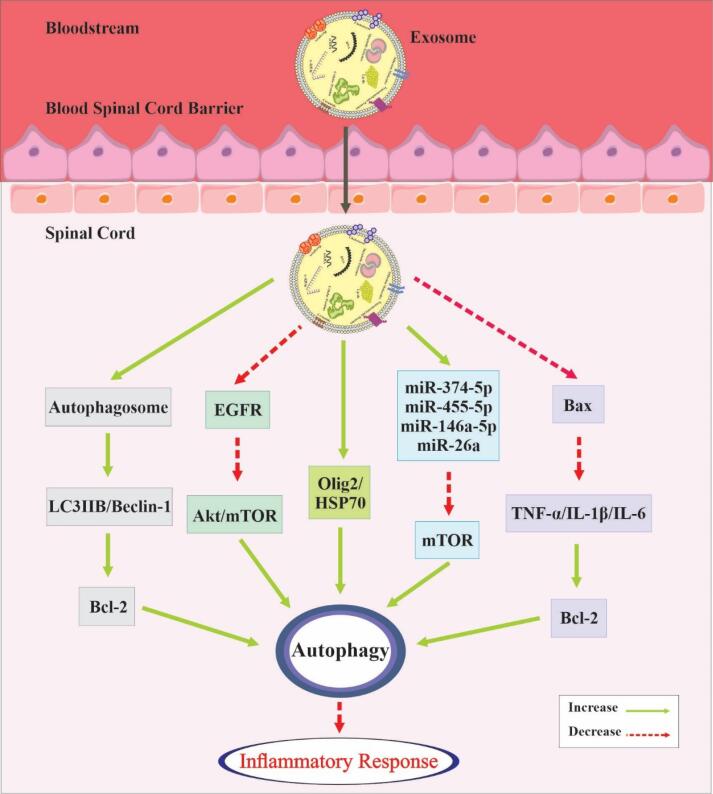
A visual representation summarizing both the effects of exosomes on autophagy and the entire autophagic process. Exosomes exhibit the capacity to cross the BSCB and form diverse associations with the autophagic pathway

 A study reported that rapamycin, an mTOR inhibitor, led to a severe reduction in exosome release by activating autophagy. Conversely, inhibition of autophagy induced by wortmannin, a phosphatidylinositol 3-kinase inhibitor, or by the removal of the autophagy-related protein ATG5 via CRISPR/Cas9, resulted in a substantial increase in exosome release.^113^ Moreover, research has indicated that ROS can regulate the mTOR/TFE3 axis, leading to autophagy dysregulation and abnormal lysosomal biogenesis. Thus, the inhibition of autophagic flux can promote exosome formation.^114^ Figure 3 summarizes both the impact of exosomes on autophagy and the entire autophagic process.

 Exosomes and autophagy are two interrelated processes that influence the cellular response to SCI. They can exchange molecules and regulate apoptosis, inflammation, and axonal preservation after SCI by activating mTOR and autophagy-related proteins.^115^ They can also deliver cargo and modify surface markers, which enhance the recovery from SCI and the maintenance of cellular homeostasis. However, the exact molecular mechanisms of their interaction are still unclear.^116^ Moreover, the optimal dose and efficacy of exosome implantation for SCI treatment are not well-established due to the complexity of their crosstalk with autophagy.^117^ Therefore, more research is needed to elucidate the underlying mechanisms and improve our understanding of this topic.^118^ Exosomes and autophagy share some molecular machinery, such as ATG proteins, ESCRT complexes, and Rab GTPases.^119^ They can also regulate each other’s activity and function through feedback loops, such as exosome-mediated inhibition of autophagy or autophagy-mediated degradation of exosomes.^120^ They can also cooperate to form amphisomes, which are hybrid vesicles that fuse with lysosomes for degradation or secretion.^121^ Exosomes and autophagy have been implicated in the regulation of cellular fitness after brain injury, synaptic pruning, and cancer therapy.^122^ They also play important roles in ocular surface and retinal diseases, which are often associated with SCI.^123^ Exosomes with higher microRNA-25 levels demonstrated enhanced neuroprotection, reducing inflammation and oxidative stress in ischemic spinal cords.^124^ They can also be used as therapeutic agents or biomarkers for ocular diseases. For example, exosomes derived from mesenchymal stem cells or retinal pigment epithelial cells can promote retinal regeneration and protect against ischemia-reperfusion injury. Autophagy inducers or inhibitors can modulate the survival and function of retinal cells under stress conditions.^125^ These findings suggest that targeting the crosstalk between exosomes and autophagy could be a promising therapeutic strategy for SCI.

## Discussion

 SCI is a serious condition that has a poor prognosis for nerve system dysfunction. The initial lesion to the spinal cord triggers neuronal loss, while long-term effects are attributed to edema, inflammation, reactive gliosis, cavitation, and dysregulated autophagy. SCI can quickly compromise the function of multiple organs, resulting in irreversible damage and loss of sensory, motor, and autonomic functions. Due to its high mortality rate and significant medical expenses, SCI has become a global health concern. Various treatment options are available for SCI, including surgical decompression, neuroprotection, methylprednisolone, magnesium, cerebrospinal fluid drainage, therapeutic hypothermia, as well as the most promising cell-based approaches and bioengineering materials. Despite the progress that has been made, treating SCI remains a significant clinical challenge. While a few current SCI therapies have shown improvements in axonal regeneration, neuroprotection, immunomodulation, and angiogenesis, the efficacy of treatments for SCI is still limited. Exosome therapy has gained significant attention in recent years. Exosomes are extracellular vesicles that have emerged as versatile regulators of various physiological and pathological processes, holding immense potential as therapeutic tools. These vesicles, derived from different cells, can modulate gene, protein, and miRNA expression in their target cells and tissues to induce neuroprotection and neurorestorative effects. Notably, exosomes derived from specific cell types have demonstrated the ability to mitigate inflammation and enhance angiogenesis, neurogenesis, and functional recovery following SCI. Modified exosomes can serve as efficient carriers of exogenous genes, proteins, and chemicals to recipient cells, thereby augmenting their therapeutic potential. Furthermore, augmenting gene and miRNA modulation in exosome-producing cells can enhance the therapeutic efficacy of exosomes.

 Studies have reported that exosomes can bind to recipient cells through conventional receptor-ligand interactions, as well as those involved in cell-cell communication, membrane fusion, and endocytosis. Exosomes have also been found to carry biologically active cytokines and inflammasome components that activate immune responses. In addition, exosomes have shown interactions with autophagy, a crucial physiological process that facilitates the efficient degradation and conversion of cytosolic components, particularly during periods of stress. Previous studies have shown a strong correlation between different autophagy pathways and the biogenesis and secretion of exosomes.^7^ The interaction between exosomes and autophagy is a promising area of research for the development of novel therapeutic strategies for SCI. For example, exosomes derived from mesenchymal stem cells have been shown to regulate autophagy in recipient cells, thereby promoting cell survival and reducing inflammation. Moreover, exosomes can carry specific miRNAs that can target various genes involved in autophagy regulation. Exosomes can also enhance the therapeutic potential of cell-based approaches, such as stem cell therapy, by promoting cell survival and modulating immune responses.

 Several studies have investigated the potential of exosomes and autophagy as treatment mechanisms for SCI.^12^ Interestingly, the processes of exosome biogenesis and autophagy, which may either assist or conflict with each other, are interconnected. After SCI, both exosomes and autophagy exhibit anti-apoptotic, anti-inflammatory, and axonal protective effects. Under physiological circumstances, autophagy and exosome secretion are in constant equilibrium. In the context of impaired autophagy, exosome production and release are increased as an alternative strategy to reduce cellular stress and maintain cellular homeostasis. Activating autophagy decreases exosome release, as the pathways for exosome production and autophagy overlap, and promoting autophagy diverts more exosomes into this pathway, thereby limiting exosome release. Also, exploring the transformation of damaged spinal cord cells into iPSCs for neural regeneration via autophagy manipulation with specific reprogramming factors (4F) reveals promise for SCI treatment, pending further validation before clinical application.

 Exosomes can carry specific components that can repair damaged cells and promote cell development, while autophagy can alter the microstructures of cells to trigger cell death. Although some results have shown potential for the treatment of certain illnesses by harnessing the interplay of exosomes with autophagy, conclusive evidence regarding the safety and effectiveness of exosome implantation is still lacking.^101^ It is assumed that autophagy and exosomes overlap biochemically depending on their relationship in SCI. Increasing evidence suggests a crucial interplay between these processes in SCI. Additionally, biomarkers for regulatory factors of both autophagy and exosome signaling have been proposed. Modulating autophagy and exosome treatment may provide several potential benefits in SCI pathological conditions, such as promoting cell survival, inhibiting apoptosis, reducing inflammation, increasing microvascular density, enhancing immune responses, and restoring motor function. The thorough investigation of the interaction between exosomes and autophagy is of great importance, specifically due to its potential applications in clinical trials. If progress is made in this field, it could have numerous therapeutic implications. However, further research is necessary to fully comprehend the mechanisms underlying the effects of autophagy and exosome treatment, and to optimize their therapeutic potential.

## Conclusion

 This review indicates that autophagy and exosome biogenesis clearly interact, influencing each other’s cellular processes via various signaling pathways. Through preventing apoptosis, regulating inflammatory responses, and maintaining axonal integrity following damage, this interaction is thought to be essential in the treatment of SCI. The review emphasizes the therapeutic potential of focusing on exosome-autophagy pathways for SCI recovery and offers a succinct summary of new signaling pathways. Additionally, after SCI, the balance between autophagic activity and exosome secretion is constantly controlled, offering new possibilities for combinatory treatment methods. Even though studies have produced encouraging findings, more investigation is required to completely clarify the underlying processes, improve therapeutic approaches, and prove safety and effectiveness in clinical settings.

## Competing Interests

 The authors have no relevant financial or non-financial interests to disclose.

## Consent to Participate

 Not applicable.

## Consent for Publication

 Not applicable.

## Data Availability Statement

 Not applicable.

## Ethical Approval

 Not applicable.
